# Supplementary material for: Causal interplay between lactose intolerance and gut microbiota: a combined bidirectional Mendelian randomization and in vivo validation study
Source: Front Nutr. 2026 Jun 1;13:1803337. doi: 10.3389/fnut.2026.1803337 (PMC13265576; doi:10.3389/fnut.2026.1803337)
Supplement: Supplementary file 1 [file Data_Sheet_1.zip › supplementary materials/Forward/leave one out cross validation/ebi-a-GCST90027502.finngen_R12_E4_LACTONAS.pdf]

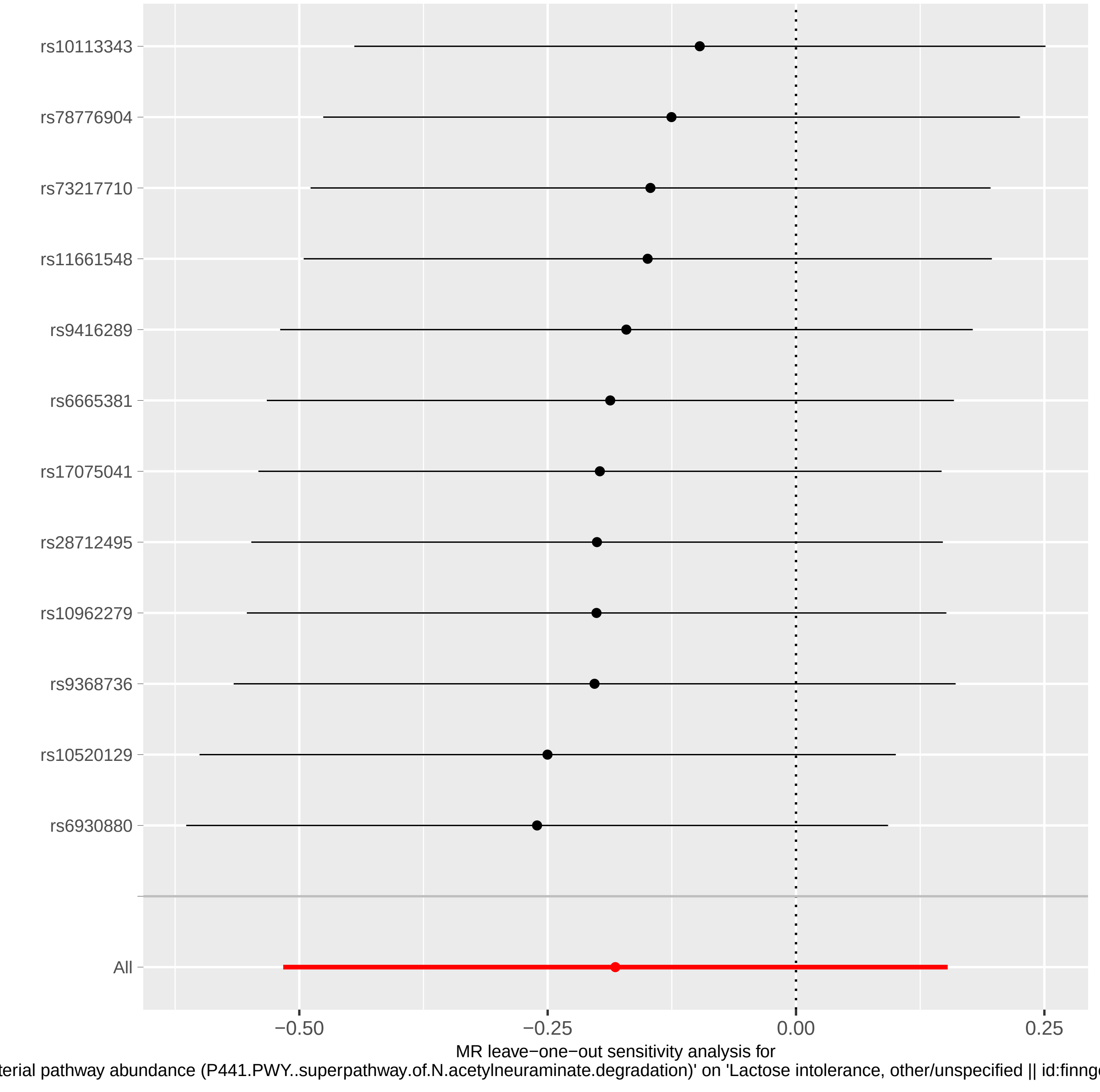
rs10113343
rs78776904
rs73217710
rs11661548
rs9416289
rs6665381
rs17075041
rs28712495
rs10962279
rs9368736
rs10520129
rs6930880
All
−0.50
−0.25
0.00
0.25
MR leave−one−out sensitivity analysis for
terial pathway abundance (P441.PWY..superpathway.of.N.acetylneuraminate.degradation)' on 'Lactose intolerance, other/unspecified || id:finng
